# Humpback Whale Populations Share a Core Skin Bacterial Community: Towards a Health Index for Marine Mammals?

**DOI:** 10.1371/journal.pone.0090785

**Published:** 2014-03-26

**Authors:** Amy Apprill, Jooke Robbins, A. Murat Eren, Adam A. Pack, Julie Reveillaud, David Mattila, Michael Moore, Misty Niemeyer, Kathleen M. T. Moore, Tracy J. Mincer

**Affiliations:** 1 Woods Hole Oceanographic Institution, Woods Hole, Massachusetts, United States of America; 2 Center for Coastal Studies, Provincetown, Massachusetts, United States of America; 3 Josephine Bay Paul Center, Marine Biological Laboratory, Woods Hole, Massachusetts, United States of America; 4 University of Hawaii at Hilo, Hilo, Hawaii, United States of America; 5 The Dolphin Institute, Honolulu, Hawaii, United States of America; 6 Hawaiian Islands Humpback Whale National Marine Sanctuary, Kihei, Hawaii, United States of America; 7 International Fund for Animal Welfare, Yarmouth Port, Massachusetts, United States of America; U.S. Geological Survey, United States of America

## Abstract

Microbes are now well regarded for their important role in mammalian health. The microbiology of skin – a unique interface between the host and environment - is a major research focus in human health and skin disorders, but is less explored in other mammals. Here, we report on a cross-population study of the skin-associated bacterial community of humpback whales (*Megaptera novaeangliae*), and examine the potential for a core bacterial community and its variability with host (endogenous) or geographic/environmental (exogenous) specific factors. Skin biopsies or freshly sloughed skin from 56 individuals were sampled from populations in the North Atlantic, North Pacific and South Pacific oceans and bacteria were characterized using 454 pyrosequencing of SSU rRNA genes. Phylogenetic and statistical analyses revealed the ubiquity and abundance of bacteria belonging to the *Flavobacteria* genus *Tenacibaculum* and the *Gammaproteobacteria* genus *Psychrobacter* across the whale populations. Scanning electron microscopy of skin indicated that microbial cells colonize the skin surface. Despite the ubiquity of *Tenacibaculum* and *Psychrobater* spp., the relative composition of the skin-bacterial community differed significantly by geographic area as well as metabolic state of the animals (feeding versus starving during migration and breeding), suggesting that both exogenous and endogenous factors may play a role in influencing the skin-bacteria. Further, characteristics of the skin bacterial community from these free-swimming individuals were assembled and compared to two entangled and three dead individuals, revealing a decrease in the central or core bacterial community members (*Tenacibaculum* and *Psychrobater* spp.), as well as the emergence of potential pathogens in the latter cases. This is the first discovery of a cross-population, shared skin bacterial community. This research suggests that the skin bacteria may be connected to humpback health and immunity and could possibly serve as a useful index for health and skin disorder monitoring of threatened and endangered marine mammals.

## Introduction

It is now recognized that microbes play an important role in the health of mammals, especially humans [1], [2]. This theme has led to the emergence of comparative studies targeting other disparate mammalian species, aiming to understand the connections between microbiota and exogenous (environmental) and endogenous (host-associated) influences [3]. The largest mammalian organ in direct contact with the environment is the skin, and the skin of marine mammals is a particularly interesting surface because it is in constant contact with seawater microorganisms, which are generally orders of magnitude more populous than airborne microorganisms surrounding terrestrial mammals [4], [5]. Additionally, seawater microorganisms can exhibit population shifts related to altered environmental conditions [6], [7] that have the potential to influence the composition of microbes residing on marine mammal skin.

Recently, the skin of humpback whales (*Megaptera novaeangliae*) were shown to associate with diverse communities of bacteria that differ from the communities present in the seawater [8]. Further, specific groups of bacteria were associated with most individuals studied, regardless of whale age or sex [8]. These findings suggest that humpback whales may harbor specific skin-bacterial associates. However, documenting if and how skin bacterial communities vary between humpbacks from different populations and geographic areas is necessary to understand the extent of this host-bacterial specificity. Importantly, documenting the presence of a core, or central, microbial community across all members of a population of marine mammals may provide an indication of a relationship that is fundamental to the host [9], [10].

The skin microbiome of humpback whales is particularly attractive for cross-population studies. This species is found in all oceans, and the structure, dynamics and ecology of many populations have been well-studied [11], [12]. Humpback whales migrate annually from mid- to high latitude feeding grounds to low latitude breeding and calving grounds [13]–[17], and some individuals hold the most extensive mammalian migration on record [18]. The ubiquity and migratory nature of this species provides an opportunity to study the impact of various oceanic conditions on the composition of the skin microbiota. Furthermore, humpback whales fast while on migration and throughout the breeding season, and so there is also an opportunity to evaluate how routine metabolic stress may affect microbial communities. Lastly, this species is vulnerable to human impacts in many parts of its range, and the effects of those impacts can be cryptic. Understanding the relation of the skin microbiome to health and geography may enhance monitoring and conservation efforts of populations.

The idea that humpback whale skin harbors specific bacterial associates may have larger implications for health or health monitoring of these cryptic animals. At first mention, it might seem nonsensical that the surface epidermis, a layer of dead skin, has any connection to health. However, in humans there is evidence suggesting links between the composition of the skin-bacteria and inflammatory, allergic conditions [19]. Also, shifts in skin-bacteria together with an altered host immune response may lead to the development of skin diseases or disorders [20]. There has been much less attention towards the study of marine mammal skin, microbes and health. Historically, microbes have been observed on the skin of odontocetes, and antibiotic production by these cells was thought to prevent colonization of the skin by pathogens [21]. Additionally, captive cetaceans exposed to water purified with oxidizing agents (chlorine or ozone - aimed to reduce microbial abundances in the seawater) resulted in the animals developing skin infections that may have been linked to a lack of beneficial microbes on their skin [22]. It is clear that an understanding of skin-bacteria on normal, healthy marine mammals is necessary before any connections about health can be explored. Importantly, this framework will be useful to recognize if and how skin-bacteria differ on unhealthy animals, including the growing number of marine mammals displaying skin lesions and disorders [23].

The goal of this study was to characterize the skin-associated bacteria from humpback whales, and explore whether animals from diverse geographic areas and populations share a core, or central, bacterial community. Additionally, this investigation examined the extent that geographic area and host-specific factors (population, age, sex and metabolic state) may contribute to variability within the skin-bacteria. Lastly, the diversity and composition of skin-bacteria from the apparently healthy animals was compared to entangled and recently deceased individuals (with potentially reduced or extinct immune responses), and used to examine the potential for skin-bacteria to provide any clues about a compromised health state.

## Materials and Methods

### Ethics Statement

Collection of skin samples was conducted under NOAA permits #633-1778, 633-1778, 932-1489, 774-1714, 1071-1770-00, 1000-1617, and the approval of the Government of American Samoa. All samples were collected according to permit guidelines.

### Samples

Skin samples were collected from two geographic areas in the North Pacific: one feeding ground (Southeast Alaska, summer 2009) and the breeding ground (Hawaiian Islands, winter 2007 and 2008) that those individuals share with others in the North Pacific. Samples were also collected from a South Pacific breeding area (American Samoa, winter 2009) and a North Atlantic feeding area (the Gulf of Maine, summer 2009) (Table S1). Skin was obtained by biopsy sampling techniques on the upper flank near the animal's dorsal fin [24]. Additionally, humpback whales shed pieces of skin from during high-energy surface behaviors [25] and these can originate from any skin surface on the body. Freshly dislodged samples were collected using nets and sieves. Marine mammals have been shown to shed the surface epidermis regularly [22], and therefore sloughed skin is likely only slightly older in age than the biopsied skin. Additionally, some samples were obtained from the suction cup used to attach acoustical tags to whales (Table S1). Skin samples were handled using sterile tools, with the exception of sloughed skin nets and sieves which were only rinsed with seawater between uses. However, skin was in contact with these nets and sieves for a very short amount of time (seconds). Skin was kept on ice for no more than 10 hours before freezing. Samples of seawater were also obtained with the Hawaii samples, and collection methods outlined previously [8]. We also requested samples from stranding and entanglement response networks to compare free-swimming whales to individuals that were potentially health-impaired. As detailed in Table S2, the samples available to this study included two live whales entangled in fishing gear and three whales sampled after death.

Data on whale age, sex, metabolic state and health state were made at the time of sampling or obtained from long-term population monitoring programs. Sex was known from molecular genetic analysis [26], visual observation of the genital slit [27], the presence of a dependent calf (for females) or sex-linked stereotypical behaviors on the breeding grounds. Exact age was known for individuals first documented as dependent calves. Individuals were considered independent juveniles from ages 1–4 and adults from 5 years onward. When exact age was not known, age class was inferred based on its minimum age or visually assessed from size and behavior. Metabolic state was determined by geographic area (for non-nursing calves), with animals in their breeding and feeding grounds displaying catabolic and anabolic metabolisms, respectively.

### Pyrosequencing of V1–V3 regions of bacterial SSU rRNA genes

Nucleic acids were extracted from 2–70 mg (generally 25 mg) of skin or 220–240 ml of surface seawater (<0.5 m) using previously described methods [8]. DNA was quantified using the PicoGreen fluorescent assay (Invitrogen Corp., Carlsbad, CA, USA) on a SpectraMax M2 plate reader (Molecular Devices Corp., Sunnyvale, CA, USA). Barcoded primers targeting the V1–V3 regions of the SSU rRNA genes, 27FB and 519R, were utilized for pyrosequencing analysis (primers and barcodes described in [28]). Triplicate 20 µl PCR reactions contained 2.5 U of Pwo SuperYield DNA polymerase (Roche Applied Science, Indianapolis, IN USA), 1× Pwo SuperYield buffer, 200 µM of each dNTPs, 200 nM of each barcoded fusion primer, and 4–12 ng of genomic template. After an initial denaturation step at 95°C for 3 min, the reaction conditions were: 30–35 cycles of 95°C for 15 s, 55°C for 30 s, and 72°C for 2 min, concluding with an extension at 72°C for 7 min. The reactions were carried out in an Eppendorf Mastercycler (Eppendorf, San Diego, CA, USA). A subset of each reaction (14 µl) was run onto a 1% agarose/TBE gel, and the replicate reactions were combined, purified using the Qiagen MinElute Gel Extraction kit (Qiagen Inc.), and quantified using the PicoGreen fluorescent assay and a Bio-Rad CXF96 Real-Time system (Bio-Rad Laboratories, Hercules, CA, USA). Barcoded amplicons were combined in two separate equimolar ratio libraries and shipped to the University of Illinois W.M. Keck Center for Comparative and Functional Genomics for sequencing. Each library was sequenced on one-half of a picotiter plate using a 454 Life Sciences Genome Sequencer FLX instrument with Titanium series chemistry (Roche, Branford, CT, USA). Sequencing was conducted from primer B using an amplicon protocol.

### Sequence processing

Processing of SFF files was conducted using Mothur [29]. After barcode and primer removal, 754,593 sequences were resolved from the combined libraries with an average length of 498 bp. Sequences were then aligned to the 16S rRNA molecule using the Silva database alignment template [30], trimmed at similar molecule locations and pre-clustered to eliminate outliers. After removal of sequencing and PCR-related noise and quality trimming [31], the total number of sequences was reduced to 536,055 with an average read length of 261 bp. An additional 10,768 sequences were found to be chimeric and removed. In order to identify non-bacterial sequences, the data were classified using first the RDP [32] and secondly using the Silva taxonomy training sets with a Bayesian classifier. Sequences identified as chloroplasts (1,885) were subsequently removed from the dataset. A distance matrix was constructed of the sequences, and sequences were clustered into operational taxonomic units (OTUs) using the average neighbor algorithm at a 97% similarity level. In order to verify the taxonomy of sequences, representative sequences from unclassified OTUs (∼2000 sequences) were aligned using the SINA web aligner and imported into a Silva 106 non-redundant database using the ARB software [33]. After aligning the sequences in the database, sequences (152) further identified as chloroplasts or mitochondria were removed, and sequences belonging to a number of candidate groups that were not included in the training set were re-classified. The finalized dataset included 522,954 sequences with an average length of 261 bp, grouped into 9,088 OTUs. In order to compare the alpha and beta diversity of samples of equal sample size, the samples were randomly sub-sampled to a depth of 3,663 sequences, which corresponded to the lowest number of sequences recovered in the dataset for an individual sample. All analyses were conducted on the sub-sampled sequence set. Raw sequence data were archived under NCBI Sequence Read Archive BioSample accessions SAMN02566578–SAMN02566647.

Phylogenetic trees were constructed separately for sequences classifying to the *Tenacibaculum* and *Psychrobacter* genera, and included the most dominant sequences from this study as well as reference sequences of recognized species from each genus and previously published environmental gene clones of high similarity. Because of the short nature of the sequences from this study, the trees were first constructed using the RAxML maximum likelihood method [34] with nearly full-length reference sequences, with rapid bootstrap analysis on 1,000 runs. The amplicon sequences obtained from this study, as well as short sequences from the bottlenose dolphin study [35] were then added to the tree without changing the topology using the ARB parsimony interactive method. All analyses were done using the ARB software [33].

The diversity of closely related organisms within the genera *Tenacibaculum* and *Psychrobacter* was further investigated using oligotyping, [36] a supervised computational method that allows the identification of closely related bacterial organisms that are grouped into one taxon [37], [38]. Oligotyping analyses were performed on 148,974 *Tenacibaculum* and 140,729 *Psychrobacter* reads using the oligotyping pipeline version 0.9 (available from http://oligotyping.org). To reduce the impact of noise, minimum substantive abundance (*M*) of an oligotype was set to 50 for both runs, as a result of which, any oligotype with most abundant unique sequence with less than 50 reads was removed from the final results. Oligotyping analysis of *Tenacibaculum* and *Psychrobacter* spp. reads resulted in 98 and 53 oligotypes, respectively. Noise removal with *M* discarded 2.00% and 0.73% of *Tenacibaculum* and *Psychrobacter* spp. reads, respectively.

### Statistical analyses

For testing differences in the bacterial community richness between samples, non-parametric comparison tests were conducted using the software Minitab version 13 (Cleverbridge, Inc, Chicago, IL). For assessing beta diversity of the bacterial community among sample groupings, a relative abundance matrix of the 97% similarity grouped OTUs were square root transformed and further analyzed using the PRIMER 6 version 6.1.13 software (PRIMER-E Ltd., Plymouth, UK) [39] using statistical tests and parameters designed for comparison of ecological datasets [40]. A distance matrix was constructed using Bray-Curtis similarity, and both nonmetric multidimensional scaling (NMS) ordination (2-dimensional representation of bacterial community structure) and hierarchical clustering analysis (CLUSTER) (similarity dendrogram) were used to explore groupings of the samples without pre-defined sample classes. PERMANOVA tests were used to test for significant differences in bacterial community composition between pre-defined sample groups, e.g., whales sampled in different geographic areas, and were conducted using a Bray-Curtis similarity matrix of relative abundance data with 9999 permutations under Type II sum of squares and a reduced model. Significance levels were confirmed using Monte Carlo simulations. When significant differences were identified between sample categories, similarity percentages test (SIMPER) was preformed to assess each OTU's contribution to a pre-defined sample group. Multidimensional scaling analysis on samples using *Tenacibaculum* and *Psychrobacter* spp. oligotypes were performed using Morisita-Horn distance measure.

### Scanning electron microscopy

Skin biopsy samples collected from seven individuals in the Gulf of Maine during 2010 and 2011 (Table S1) were preserved in a 2% glutaraldehyde and 2% formalin solution in phosphate buffered saline (PBS) and stored at 4°C. In preparation for microscopic analysis, ∼30 mg samples containing the skin surface and several mm below were rinsed at room temperature 3× in PBS solution for 20 min, followed by 10 min in a 50% ethanol and PBS solution, and stored overnight in a 70% ethanol and PBS solution. The following morning, samples were rinsed for 10 min each in 85% ethanol and PBS and 95% ethanol and PBS. Samples were subsequently rinsed 3× for 15 min each in ethanol. Samples were then dried to the critical point using a Samdri 780A (Tousimis Research Corporation, Rockville MD, USA) and sputter-coated with a 10 nm coat using a Leica EM QSG100 Modular High Vacuum Coating System (Leica Microsystems, Buffalo Grove, IL, USA). Samples were viewed on a JEOL 840 scanning electron microscope (JEOL, Peabody, MA, USA) with representative images captured.

## Results

### Richness of whale skin-associated bacteria is similar across geographic areas

**Figure 1:**
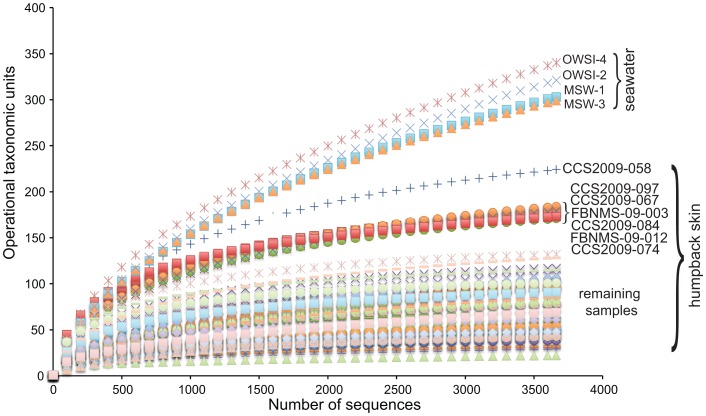
Rarefaction analysis of bacterial SSU rRNA gene sequences. This analysis represents data subsampled at 3,662 sequences per sample. Samples with OTU richness greater than 130 are listed by name.

SSU rRNA gene pyrosequencing data were used to examine the skin-bacteria from the normal (apparently healthy) whales. Rarefaction analysis of the 3,662 SSU rRNA gene sequences per sample revealed that the majority of the whale-skin bacterial communities reached a plateau at <100 OTUs (Figure 1). The average observed number of bacterial OTUs associated with whale skin varied among 71–99 with some variation among samples (Table 1). There was no significant difference in the richness of the skin-bacterial communities (measured by OTU abundance) among geographic areas (Mood's Median Test, chi-square = 2.92, df = 3, p = 0.404), and other measurements of diversity and evenness were also comparable (Table 1). OTU abundance from all of the humpback samples was significantly less than seawater (Mann Whitney Test, W = 2346, p<0.01, Table 1, Figure 1), suggesting that factors shaping the skin and seawater microbial communities are distinct.

**Table 1:** Observed OTUs (97% similarity grouped) and diversity indices from 3,662 sequences for the different sample categories (data for normal animals only).

Samples (samples, individuals)	OTUs (St. Dev.)	OTU Range	Shannon Diversity (H′) (St. Dev.)	Simpson Diversity (1−λ′) (St. Dev.)	Pielou's evenness (J′) (St. Dev.)
Seawater (4 s.)	316 (19)	299–340	3.021 (0.27)	0.805 (0.06)	0.550 (0.004)
Gulf of Maine (21 s., 19 ind.)	99 (56)	32–224	2.638 (0.64)	0.850 (0.10)	0.592 (0.09)
Hawaii (18 s., 14 ind.)	71 (33)	22–132	2.192 (0.95)	0.717 (0.24)	0.521 (0.193)
American Samoa (13 s., 12 ind.)	90 (46)	19–179	2.384 (0.74)	0.805 (0.11)	0.544 (0.11)
Alaska (5 s., 3 ind.)	72 (19)	51–86	2.058 (0.67)	0.717 (0.24)	0.471 (0.14)

### Composition of skin-bacterial community in healthy individuals is primarily related to geographic area, metabolic state and sample type

**Figure 2:**
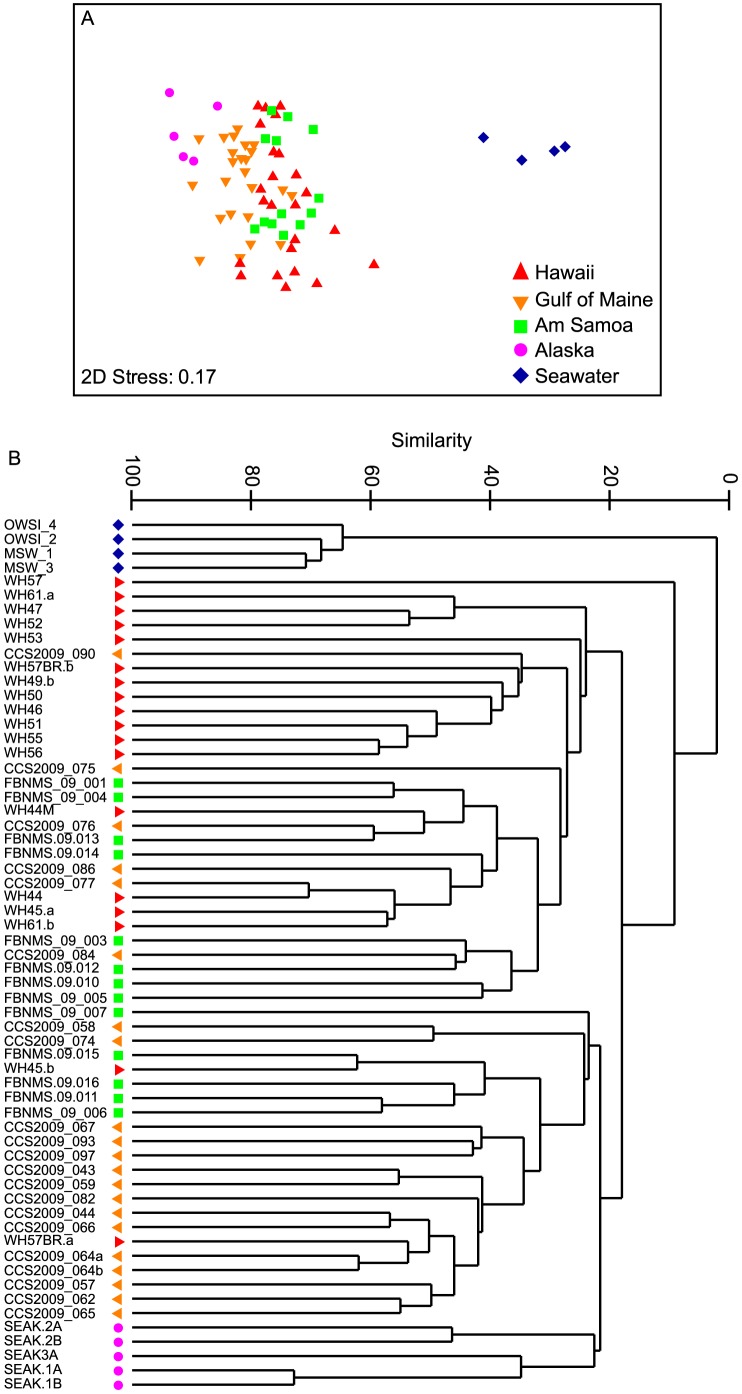
Similarity analysis of whale skin-bacteria. (A) Clustering dendogram, and (B) nonmetric multidimensional scaling analysis of 97% similarity grouped OTUs of bacterial communities associated with whale skin sampled from different populations and locations, and seawater.

Nonmetric multidimensional scaling analysis comparisons of the 97% similarity grouped OTUs revealed that skin-bacteria generally grouped by geographic area, and were highly similar in composition compared to the surface seawater samples (Figure 2a). Clustering analysis of the OTUs further revealed that the similarity between the whale skin-bacterial samples was broad, ranging from 10–65% (Figure 2b). Skin taken from whales in Hawaii, Gulf of Maine and Alaska each generally comprised a distinct cluster (Figure 2b). PERMANOVA with pair-wise tests revealed that skin-bacteria between all sampling locations were significantly distinct, except for American Samoa and Hawaii (Table 2).

**Table 2:** PERMANOVA analysis of the effect of geographic area, metabolic state, age class, sex and sample type on the 97% OTU grouped bacterial SSU rDNA data from humpback skin (GOM = Gulf of Maine).

Data	Variation	d.f.	SS	Pseudo-F^f^ or t-value^t^	p-value
Normal whales	Location (Hawaii, GOM, Alaska, Am Samoa)	3	20279	2.9121^f^	0.001***
Hawaii vs. GOM				1.5052^t^	0.006**
Hawaii vs. Am Samoa				1.2228^t^	0.073
Hawaii vs. Alaska				1.4257^t^	0.004*
GOM vs. Am Samoa				1.7878^t^	0.001***
Am Samoa vs. Alaska				2.1007^t^	0.001***
Normal whales	Sample type (biopsy, sloughed, tag)	2	15231	3.2809^f^	0.001***
Biopsy vs. sloughed				2.2608^t^	0.001***
Biopsy vs. tag				1.1763^t^	0.123
Sloughed vs. tag				1.1683^t^	0.150
Normal whales	Age (calf, juvenile, adult)	2	7217.8	1.4042^f^	0.065
Calves vs. juveniles				1.0371^t^	0.342
Calves vs. adults				1.1616^t^	0.141
Juveniles vs. adults				1.3665^t^	0.028*
Normal whales	Metabolic state (anabolism, catabolism)	1	12670	3.6307^f^	0.001***
Normal whales	Sex (male, female)	1	2444.1	0.90575^f^	0.513

***p≤0.001,

**p≤0.01,

*p≤0.05.

**Figure 3:**
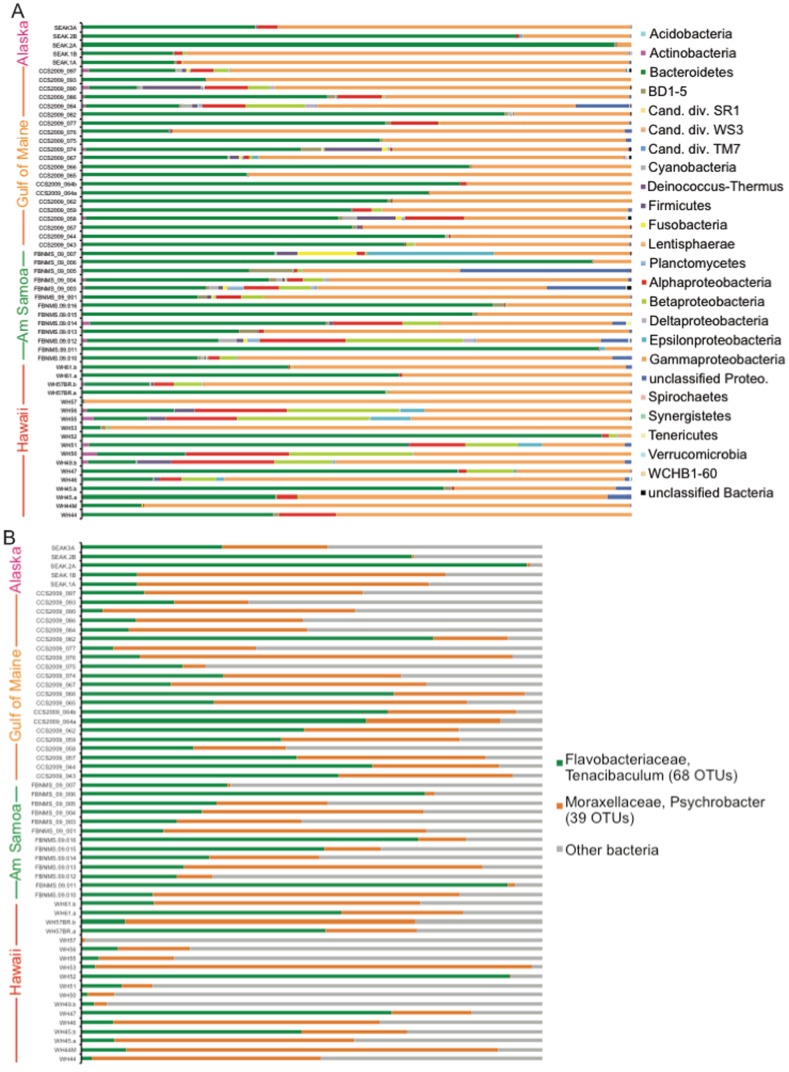
Taxonomic composition whale skin-bacteria. The 97% similarity grouped OTU bacterial communities were associated with humpback whale skin sampled from different populations and locations. Graphs represent total composition (A), and abundances of skin-bacterial sequences specifically belonging to the *Tenacibaculum* and *Psychrobacter* lineages (B). Each bar graph represents the relative abundance of each major taxonomic group, and sums to 100%.

Phylogenetic identifications of the bacterial SSU rDNA OTUs demonstrated that 23 major taxonomic groups associated with the skin of healthy humpback whales (Figure 3a). The majority of whale skin samples were associated with bacteria belonging to the *Bacteroidetes* and *Gammaproteobacteria*, and many skin samples also contained bacteria belonging to the *Firmicutes* and *Alphaproteobacteria*. Two genera, *Tenacibaculum* (*Bacteroidetes*) and *Psychrobacter* (*Gammaproteobacteria*) were found to be abundant within the majority of whale skin samples (Figure 3b), and collectively made up a large portion, 55–75%, of the skin-bacterial community (Table 3). SIMPER analysis showed that dissimilarities in the abundance of the different *Tenacibaculum* and *Psychrobacter* spp. OTUs, as well as several other bacterial groups, contributed to the differences in the skin-bacteria of whales from different geographic areas (Table S2). SIMPER analysis also demonstrated that the locations with more similar skin-bacteria (American Samoa and Hawaii) harbored sequences affiliated with *Cardiobacteria* and *Aquabacterium* spp., which were not present in the results of samples from the other locations (Table S2). Animals sampled in American Samoa and Hawaii were not actively feeding (except for one nursing calf which were not included in the analysis), and therefore their metabolism is catabolic compared to the anabolic (feeding) metabolism of whales in Alaska and the Gulf of Maine. This metabolic difference was found to correspond significantly to the composition of the skin-bacteria, regardless of geographic area (Table 2). The differences in skin-bacteria related to metabolic state were largely attributed to different sequence variants of *Tenacibaculum* and *Psychrobacter* spp. (in addition to some other taxa, Table 4).

**Table 3:** Abundances of whale skin-bacterial sequences corresponding to the *Tenacibaculum* and *Psychrobacter* genera.

Genus	Range	Mean (St. dev)
Alaska		
*Tenacibaculum*	12–97%	44% (38)
*Psychrobacter*	0.4–67%	31% (32)
*Tenacibaculum+Psychrobacter*	53–98%	75% (16)
Gulf of Maine		
*Tenacibaculum*	5–76%	35% (23)
*Psychrobacter*	5–81%	36% (17)
*Tenacibaculum+Psychrobacter*	27–96%	71% (23)
American Samoa		
*Tenacibaculum*	15–92%	39% (26)
*Psychrobacter*	0.7–67%	27% (24)
*Tenacibaculum+Psychrobacter*	28–94%	65% (21)
Hawaii		
*Tenacibaculum*	0.1–93%	22% (28)
*Psychrobacter*	0.3–95%	33% (29)
*Tenacibaculum+Psychrobacter*	0.8–98%	55% (33)

**Table 4:** Results of SIMPER analysis describing 97% similarity grouped OTUs contributing to the differences between the whales with anabolic and catabolic metabolisms (>1% contribution only).

OTU	Av. abundance anabolic whales	Av. abundance catabolic whales	Average Diss.	Diss. S.D.	Contribution to differences (%)	Taxonomy
2	9.14	14.22	8.11	0.86	9.39	*Gammaproteobacteria, Psychrobacter*
3	10.61	4.31	5.78	0.90	6.69	*Bacteroidetes, Tenacibaculum*
1	4.39	7.44	5.37	0.49	6.21	*Bacteroidetes, Tenacibaculum*
4	4.23	5.83	4.10	0.63	4.75	*Bacteroidetes, Flavobacteriaceae*, uncultured humpback group
8	6.24	0.02	3.12	0.37	3.61	*Bacteroidetes, Tenacibaculum*
12	2.88	5.13	3.08	0.55	3.57	*Bacteroidetes, Tenacibaculum*
10	4.05	2.34	2.70	0.56	3.13	*Bacteroidetes, Tenacibaculum*
27	1.49	5.06	2.65	0.75	3.06	*Bacteroidetes, Tenacibaculum*
9	3.10	3.56	2.59	0.73	2.99	*Gammaproteobacteria, Moraxellaceae*
7	1.65	4.19	2.35	0.64	2.72	*Gammaproteobacteria, Psychrobacter*
19	4.26	0.16	2.10	0.57	2.43	*Gammaproteobacteria, Psychrobacter*
11	1.70	3.24	2.07	0.47	2.40	*Gammaproteobacteria, Psychrobacter*
21	3.61	0.01	1.81	0.40	2.09	*Gammaproteobacteria, Psychrobacter*
14	0.96	2.85	1.81	0.34	2.09	*Gammaproteobacteria, Psychrobacter*
15	0.70	3.11	1.61	0.71	1.87	*Betaproteobacteria, Aquabacterium*
28	3.12	0.34	1.60	0.74	1.85	*Gammaproteobacteria, Psychrobacter*
5	2.26	1.60	1.53	0.41	1.78	*Gammaproteobacteria, Cardiobacteria*
24	0.00	2.87	1.43	0.19	1.66	*Gammaproteobacteria, Acinetobacter*
29	2.81	0.30	1.38	0.61	1.60	*Bacteroidetes, Tenacibaculum*
41	1.44	1.47	1.29	0.50	1.50	*Bacteroidetes, Flavobacteriaceae*
22	0.51	2.00	1.17	0.39	1.35	*Proteobacteria*, Incertae Sedis (humpback-specific)
37	0.39	1.84	1.02	0.43	1.18	*Bacteroidetes, Flavobacteriaceae*
20	1.30	1.10	0.97	0.67	1.12	*Alphaproteobacteria*, Roseobacter clade AS-21
33	1.88	0.08	0.93	0.60	1.08	*Gammaproteobacteria, Colwellia*

Significant differences were also evident between the bacterial communities on sloughed skin versus biopsy samples (Table 2), which may be related to the anatomical locations from which the samples originated. Biopsies were generally focused on the flank whereas sloughed skin was from unknown locations on the animal. With the exception of dependent calves, samples from juveniles harbored significantly different bacteria from adults (Table 2). No relationship was found between whale sex and skin-bacteria.

### *Tenacibaculum* and *Psychrobacter* spp. are core members of the humpback skin bacterial community, but exhibit regional-specific sequence variations

**Figure 4:**
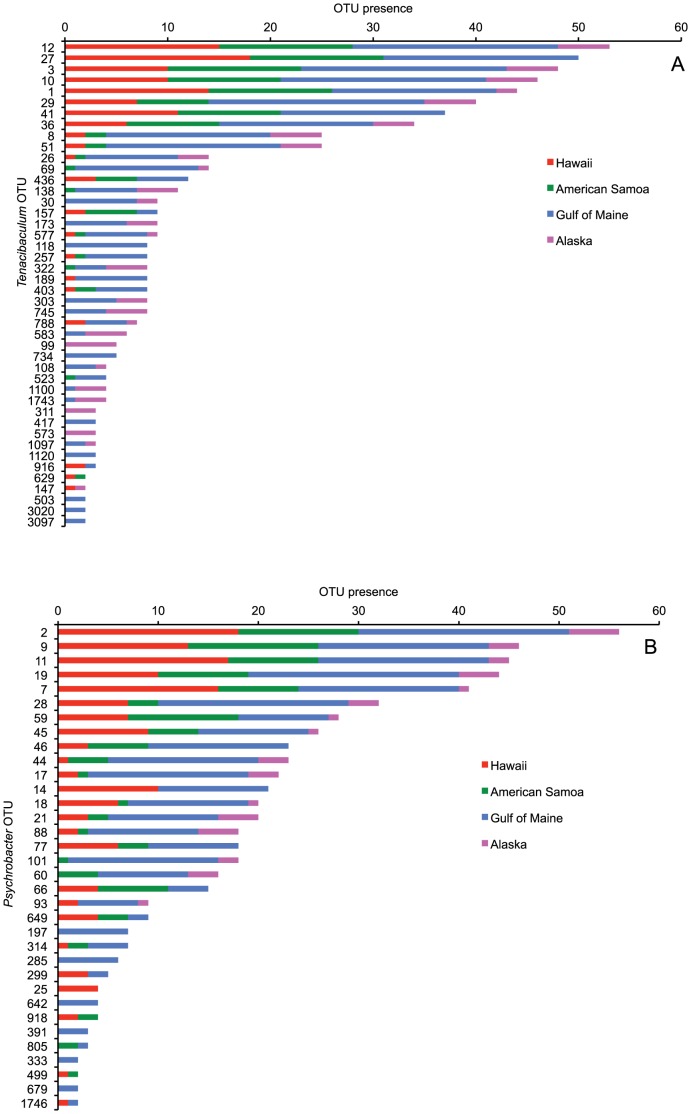
Abundances of *Tenacibaculum* and *Psychrobacter*-affiliated sequences. Presence of skin-bacterial OTUs (97% similarity grouped) classified as *Tenacibaculum* (A) and *Psychrobacter* (B), in relation to the geographic region of the whales.

**Figure 5:**
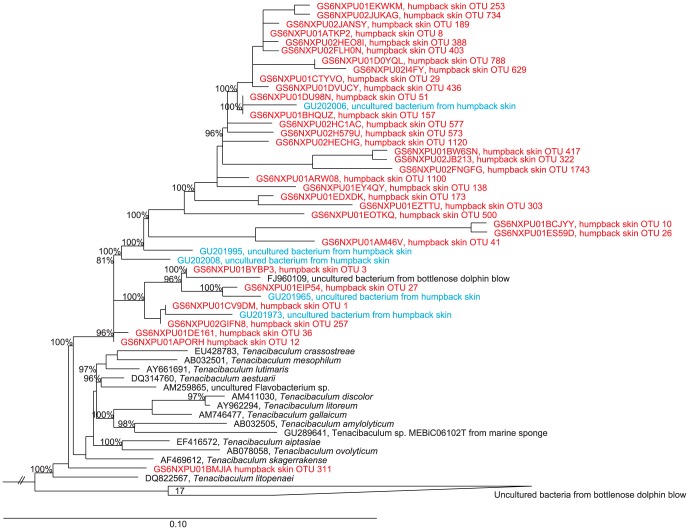
Phylogenetic relationship of *Tenacibaculum*-affiliated sequences. Represented SSU rRNA gene sequences include *Tenacibaculum*-affiliated sequences recovered from whale skin, cultivated *Tenacibaculum* isolates, and relatives within the *Tenacibaculum* lineage. Red sequences are short-reads from this study and blue are full-length sequences from a previous study on humpback whale skin [8]. The scale bar corresponds to 0.10 substitutions per nucleotide position, and only bootstrap values >70 are listed. Sequences from *Caldilinea aerophila* (AB067647), *Roseiflexus castenholzii* (CP000804) and *Actinomyces oris* (GQ421308), were used to form the outgroup. Full accession numbers for the dolphin respiratory-bacteria sequences are available in [35].

Sequences affiliated with the genus *Tenacibaculum* accounted for average abundances of 44, 35, 39 and 22% of the whale-skin communities from Alaska, Gulf of Maine, American Samoa and Hawaii, respectively (Table 3). In total, 44 OTUs (at 97% similarity grouping; represented in two or more samples) comprised this group, with the most cosmopolitan OTU present in 53 of the 57 samples from different regions (OTU 12, Figure 4A). Five of the OTUs (27, 41, 436, 257 and 403) were present in all samples except those from Alaska. A phylogenetic analysis demonstrated that the sequences from these OTUs clustered into several distinct lineages, and are related to sequences recovered from the blowholes of bottlenose dolphins [35] (Figure 5).

**Figure 6:**
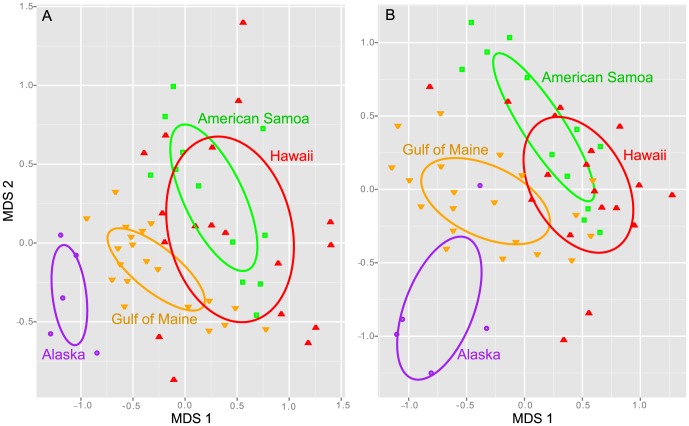
Oligotyping analysis of major humpback skin-bacterial groups. MDS analysis of the healthy animals from four regional groups based on *Tenacibaculum* (A) and *Psychrobacter* (B) oligotypes. Centroid and shape of each ellipse is defined by the distribution and standard deviation of points in the defined regional groups.

Oligotyping analysis was used to examine more subtle variations with the SSU rRNA gene of the *Tenacibaculum* spp. sequences. This analysis identified 98 *Tenacibaculum* spp. oligotypes in the whales, which differed significantly by region, but with the most overlap between samples from Hawaii and American Samoa (Figure 6A). Similar to the trends for the entire community, PERMANOVA analysis revealed that the *Tenacibaculum* spp. oligotypes differed in composition with the distinct metabolic states of the whales (Table 5). Additionally, the *Tenacibaculum* spp. oligotypes differed by sample type (biopsy or sloughed skin), suggesting that skin age and/or anatomical location of the skin plays a role in shaping these communities (Table 5).

**Table 5:** PERMANOVA analysis of the effect of geographic area, age, sex and sample type on *Tenacibaculum* and *Psychrobacter* spp. populations determined by oligotyping.

Data	Variation	d.f.	SS	Pseudo-F^f^ or t-value^t^	p-value
*Tenacibaculum*					
Normal whales	Location (Hawaii, GOM, Alaska, Am Samoa)	3	24929	3.6248^f^	0.001***
Hawaii vs. GOM				1.7933^t^	0.001***
Hawaii vs. Am Samoa				1.4839^t^	0.012*
Hawaii vs. Alaska				1.5359^t^	0.008**
GOM vs. Am Samoa				2.4447^t^	0.001***
Am Samoa vs. Alaska				2.4634^t^	0.001***
GOM vs. Alaska				1.8131^t^	0.001***
Normal whales	Sample type (biopsy, sloughed, tag)	2	13077	3.8521^f^	0.001***
Biopsy vs. sloughed				1.8692^t^	0.002**
Biopsy vs. tag				1.045^t^	0.361
Sloughed vs. tag				1.5504^t^	0.012*
Normal whales	Age (calf, juvenile, adult)	2	4727.2	1.0917^f^	0.360
Normal whales	Metabolic state (catabolic, anabolic)	1	15172	5.706^f^	0.001***
Normal whales	Sex (male, female)	1	3027.6	1.3518^f^	0.165
*Psychrobacter*					
Normal whales	Location (Hawaii, GOM, Alaska, Am Samoa)	3	13889	3.5707^f^	0.001***
Hawaii vs. GOM				1.4225^t^	0.012*
Hawaii vs. Am Samoa				1.7009^t^	0.002**
Hawaii vs. Alaska				1.5197^t^	0.01**
GOM vs. Am Samoa				2.8567^t^	0.001***
Am Samoa vs. Alaska				2.1002^t^	0.001***
GOM vs. Alaska				2.095^t^	0.001***
Normal whales	Sample type (biopsy, sloughed, tag)	2	4854	1.1335^f^	0.274
Normal whales	Age (calf, juvenile, adult)	2	4778.1	1.2284^f^	0.207
Normal whales	Metabolic state (catabolic, anabolic)	1	15867	6.349^f^	0.001***
Normal whales	Sex (male, female)	1	2761.8	1.2785^f^	0.198

***p≤0.001,

**p≤0.01,

*p≤0.05.

**Figure 7:**
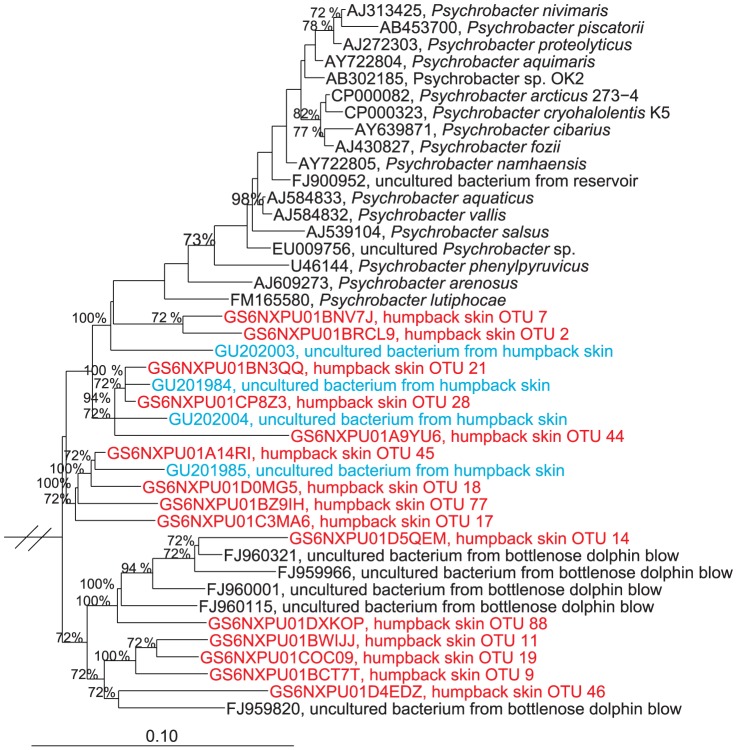
Phylogenetic relationship of Psychrobacter-affiliated sequences. Represented SSU rRNA gene sequences include *Psychrobacter*-affiliated sequences recovered from whale-skin, cultivated *Psychrobacter* isolates, and relatives within the *Gammaproteobacteria*. Red colored sequences are short-reads from this study, and blue full-length sequences are from a previous study [8]. The scale bar corresponds to 0.10 substitutions per nucleotide position, and only bootstrap values >70 are listed. Sequences from *Hymenobacter soli* (AB251884), *Salegentibacter flavus* (AY682200) and *Ureaplasma urealyticum* were used to form the outgroup.

Sequences within the *Psychrobacter* genus were present at average abundances of 31, 36, 27 and 33% of the skin bacterial communities from Alaska, Gulf of Maine, American Samoa and Hawaii, respectively (Table 3). There were 34 OTUs within the *Psychrobacter* (at 97% similarity grouping; represented in two or more samples), and six OTUs (46, 14, 77, 66, 649 and 314) were present in all samples except those obtained from Alaska (Figure 4B). The *Psychrobacter* spp. sequences grouped into several distinct phylogenetic lineages, and were also related to sequences recovered from dolphin blow (Figure 7). Oligotyping analysis of the sequence variations within the *Psychrobacter* spp. OTUs revealed 53 distinct sequence types, and these were found to differ significantly by geographic location as well as metabolic state (Table 5). As seen for *Tenacibaculum* spp., the *Psychrobacter* spp. oligotypes were most similar for whales sampled in American Samoa and Hawaii (Figure 6B).

### Microscopic visualization of skin-bacteria

**Figure 8:**
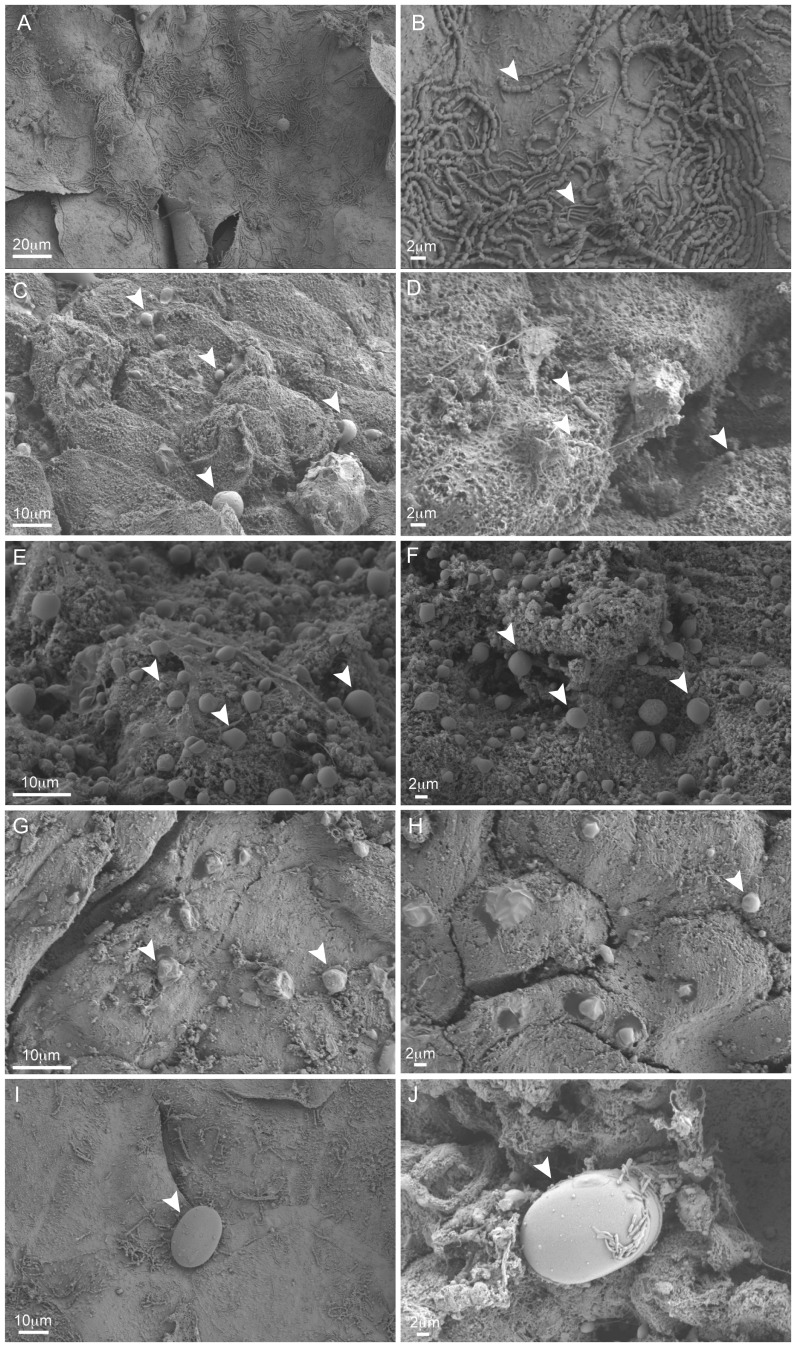
Scanning electron micrographs of whale skin-bacteria. Overview (A, C, E, G) and detailed (B, D, F, H) scanning electron micrographs of the surface of humpback whale skin from individuals CCS2010-100 (A, B), CCS2010-99 (C,D), CCS2010-96 (E,F) and CCS2010-98 (G,H). Diatoms, and microbes residing on these cells, were present on the surfaces of CCS2010-100 (I) and CCS2010-97 (J). Arrows indicate possible unique microbial cell morphotypes.

In a subset of skin samples (n = 7), microbial sized cells were identified on the skin surface using scanning electron microscopy. Variations within the density and morphologies of cells did vary between samples (Figure 8). Additionally, differences in the ‘smoothness’ of the skin, including presence of organic-matter type flocking material as well as brittleness of the skin was also evident between samples. In some samples, phytoplankton-sized cells including diatoms were visible and appeared to harbor their own smaller microbial associates (Figure 8). These results suggest that these microbes were viable and actively adhered to the skin surface, and were at least able to stay attached during the extensive sample handling and processing.

### Using the core bacterial community concept to develop a health-index for whale skin

The bacterial communities associated with the normal individuals were compared to determine indicator criteria for normal, ‘healthy’ skin, including: OTU richness of the entire community and abundances of the main core bacterial community members (*Tenacibaculum* spp.+*Psychrobacter* spp.) (Table 6). These criteria were compared to samples obtained from a small set of entangled and/or dead individuals (Table S2). In the latter animals, OTU richness and the percentage of the core bacterial community sequences fell outside of the range of the normal animals, but there were no consistent trends. Several entangled and dead individuals also harbored pathogenic-type bacteria, including members of the *Clostridium* spp., *Lachnospiraceae* spp., and *Staphylococcus* spp. and the predatory bacteria, *Bdellovibrio* spp., that were not detected in the skin samples from free-swimming animals (Table 6).

**Table 6:** Comparison of skin-bacteria among normal, health-compromised and dead humpback whales, (n/a) indicates not applicable.

Sample name (# samples or replicates)	Condition	Number of bacterial OTUs	Abundance of core bacteria (*Tenacibaculum*+*Psychrobacter* spp.)	Bacteria not detectable in healthy individuals
Normal, all populations (57)	Normal	71–99	55–75%	n/a
IFAW10-188Mn (2)	Dead	35, 51	2%, 17%	*Clostridium* spp.
Ent1 (2)	Entangled,	159, 172	44%, 32%	*Lachnospiraceae* spp., undescribed *Clostridia*, *Bdellovibrio* spp.
SxMn1023 (1)	Dead	28	90%	none
Ent2 (1)	Entangled	93	36%	*Staphlococcus* spp., *Deferribacterale*, SAR406
Calf (3)	Dead	55, 111, 130	50%, 60%, 70%	none

## Discussion

### *Tenacibaculum* and *Psychrobacter* spp. are cosmopolitan associates of whales

Humpback whales from three oceanic populations and four distinct geographic areas were found to exhibit *Tenacibaculum* and *Psychrobacter* spp. on their skin. These sequences appear to be specific to humpbacks, and are secondly related to sequences recovered from dolphin blow. The dolphin blow interacts with the blowhole epidermis and thus it is possible that dolphins also harbor specific strains of *Tenacibaculum* and *Psychrobacter* on their skin. The role or interactions of these microorganisms with humpback skin, and possibly with other marine mammals, is of considerable interest as their interactions may be linked to host health.

Bacteria within the genus *Tenacibaculum* have been previously isolated from marine habitats [41]–[44] and a variety of marine animals [45], [46] including diseased fish [47]–[49]. It is possible that humpback-specific *Tenacibaculum* spp. may be particularly adapted to a host-associated lifestyle, but their prominence (in both diversity and abundance) on healthy skin indicates an unlikely role in disease. One study did demonstrate a predatory function for *Tenacibaculum* spp. [50], and this feature might be aiding the whales in removal of fouling microbes.

Members of the genus *Psychrobacter* are generally a widespread and evolutionarily successful group of organisms. Sequences belonging to this genus have most commonly been recovered from cold environments including Antarctic soil, sea ice and deep-sea environments, and the genus is generally referred to as psychrophilic and osmotolerant [51], [52]. Additionally, sequences have been found associated with krill and ascidians [53], [54]. One possible reason that *Psychrobacter* spp. are so prevalent on humpback skin may be related to their tolerance of the ‘extreme’ type conditions of the skin surface. In the course of a single year, these animals are exposed to a large temperature range, on the order of ∼6–25°C between their seasonal feeding and breeding areas. As noted previously, some migrations are the longest of any known mammal [18], and likely create a stressful type environment for survival of many bacteria. The genome of *P. arcticus* strain 273-4 does provide genetic evidence for survival under cold and stress conditions, including changes in membrane composition and synthesis of cold shock proteins [55], and it is possible that humpback-associated *Psychrobacter* spp. also possess these qualities.

A recent metagenomic study focused on the functional-potential of the human skin-associated bacteria revealed that the resident bacteria exploited compounds produced by the skin (sugars and lipids) [56]. The same study also suggested that these bacteria might be contributing to host health by aiding in the regulation of skin acidity and epidermal permeability. Investigations into the genomic potential of *Tenacibaculum* and *Psychrobacter* spp. associated with whales could aid in understanding if human and whale skin bacteria exhibit similar functions, as well as help in revealing the potential for whale-specific interactions.

### Geographic area and/or metabolic state are related to major bacterial community differences, and more subtle variations within *Tenacibaculum* and *Psychrobacter* spp. populations

Geographic area affected the structure of the skin-bacterial community, and also drove more subtle differences in the structure of *Tenacibaculum* and *Psychrobacter* spp. populations. Water temperature as well as seawater nutrients typically vary between the subtropical (Hawaii, American Samoa) and mid- to high-latitudes (Alaska, Gulf of Maine) examined in this study, and these environmental differences may impact skin-bacteria. The structure of the bacterial community was most similar between animals sampled in Hawaii and American Samoa, and it appears that a feature of the oligotrophic tropical water, and/or a change in animal physiology, may be related to this trend. The animals from Hawaii and American Samoa exhibited catabolic metabolisms at the time of sampling. Similar shifts in microbes have been associated with changes in metabolic state and nutrient flux in other animals, but have previously only been observed in the gut microbiome [57]. For the whales, these catabolic periods may be associated with physiological-based factors previously observed in humans, which may directly affect the skin. These include decreased repair and replacement of the skin cells (and other cells with typically high turnover), decreased production of immune-related cells as well as decreased adiposity, and increased blood pressure [58]. These catabolic states may also be related to decreased wound healing [59]. The connection between metabolism, skin-bacteria and wound healing is particularly important for marine mammals due to the high incidences of lesions reported in many populations [23]. For example, it is possible that wound healing is prolonged when animals are in their breeding grounds. In this scenario, skin bacteria producing antibiotics or other defenses may be critical for protection of the whales.

This study also revealed a relationship between sample type (biopsy and sloughed skin) and bacterial community composition. This finding could be linked to the fact that the biopsied skin contained epidermis and dermis down to just before the blubber, whereas the sloughed skin was more representative of surface dead skin (stratum corneum). However, the stratum corneum does appear to be a major location in which the bacteria colonize. Anatomical location may also play a role in structuring the bacterial community. The biopsy samples were all taken from the same location on the animal (near dorsal fin), whereas the sloughed skin was removed during breaching activities from unknown locations on the animal. Repeated skin samplings of different anatomical locations from captive animals may be necessary to fully resolve the more subtle influences of anatomy on skin-bacteria. It should also be noted that the nets used to collect the sloughed skin were not sterilized prior to sampling and therefore some bacterial cells may have been transferred among samples or from the environment. Future studies using sloughed skin should consider net sterilization or single-use samplers in order to minimize the potential for cross-contamination.

### Organization of microbial cells on whale skin

In contrast to the SSU rDNA data that revealed a bacterial community dominated by two major genera, scanning electron micrographs of the humpback whale skin surface revealed large differences in the densities and morphology of surface-associated microbes. Although all samples were treated equally, it is possible that some cells as well as organic matter were dislodged during the extensive washing and processing of these tissues. Due to the rapid turnover rate of marine mammal skin, it is conceivable that recently dead versus older skin harbor different properties, that may have also affected cell adhesiveness during processing.

It is not known if the humpback-associated bacterial cells are only superficially associated, or if they are found deeper into their thick epidermis. Histological analysis of sectioned tissues may further reveal cells embedded within the epidermis. Human skin primarily supports microbial cells on the epidermis, but microbes are also found within the dermis, including hair shafts, sweat glands and sebaceous glands [60]. The specific structure of humpback skin has not been previously studied, but the epidermis of other species of whales are 15–20 times thicker than humans and excessively smooth, thus providing barrier properties to the environment, as well as limiting the attachment by microbes and penetration by pathogens [22], [23], [61], [62]. Additionally, marine mammal skin is thought to provide an innate barrier as well as programmed immune protection [63]. These features collectively suggest that bacteria are most likely restricted to the epidermal surface.

### Trends from the core bacterial community aid development of an index for ‘normal’ health

The identification of *Tenacibaculum* and *Psychrobacter* spp. as central and cosmopolitan (or ‘core’) members of the humpback skin bacterial community may aid in the development of a gauge for ‘normal’ skin health for these animals. Our results represent a wide demographic sampling of humpback whales with no obvious health impairment. It indicates some notable differences from live entangled and dead humpback whales, although available sample sizes are small. Entanglement can cause acute death, as well as chronic health effects from injury, infection and impaired mobility [64]. The live entangled whales in this study were thought to have been entangled for an extended period and exhibited mild external signs of impairment in large whales, such as pale skin, a proliferation of cyamids (whale lice) and diminished body condition [65], [66]. The differences in skin bacteria from free-swimming whales also suggest an altered health state, although we cannot exclude the possibility that bacteria were introduced and exacerbated by the foreign materials entangling the whales. Skin-bacteria from dead animals also differed from the trends reported for the free-swimming animals. Most striking was the appearance of pathogenic and predatory bacteria such as *Clostridia* and *Bdellovibrio* spp. in the deceased and entangled individuals. The emergence of these bacteria is probably related to the decay of the skin as well as a likely decrease in host immune functions and possibly also the alteration in body temperature. Interestingly, these animals tended to harbor fewer of the core bacterial community members (compared to normal animals), which further suggests that host-controlled features are probably related to the presence of these possible mutualistic or commensal populations. In laboratory studies, an alteration in the abundance of the major skin-bacterial associates has been demonstrated in mice experiencing skin disorders [67]. The microbe-host dysbiosis model introduced by Scharschmidt and Fischbach [20] explains how an imbalance in the composition of the skin microbes (perhaps caused by exogenous factors) can lead to an alteration of the host immune response. Alternatively, there may be host-susceptibility factors that cause an alteration of the host immune response (e.g., excess inflammation), which in turn triggers an endogenous shift in the skin-microbial community. Changes in both microbiota and immune response could cause a negative feedback cycle, in which both parameters become even more significantly altered.

Sightings of skin lesions on many species of marine mammals appear to be increasing, with possible correlations to changes in the marine ecosystem including pathogens [23]. This skin surface barrier and its associated core bacterial community may be an important component to maintaining health and preventing lesion formation. However, many features of this microbial association remain uncharacterized. Future studies examining the metabolism and antibiotic production of widespread *Tenacibaculum* and *Psychrobacter* spp. associates on humpbacks are needed, as well as determining whether these bacteria are more broadly found on other species of marine mammals. Additionally, examining how these cells are able to maintain residence on humpback skin is important for a broader understanding of the features that pathogen or lesion-associated bacteria may use to preserve their invasion. Finally, future studies of animals with skin lesions or disorders and other characterized health and inflammation disorders will greatly advance our understanding of the connection between the skin microbiota and health. Some of these future studies may have broader implications for mammalian skin health, including humans, and offer an opportunity to study skin-microbial adaptations under exposure to remarkably diverse environmental conditions. Overall, understanding the connections between the skin surface bacterial communities, lesion formation and animal health may aid our conservation efforts of these important animals in a time of unprecedented change to the ocean environment.

## Supporting Information

Table S1 **Summary of humpback whale tissue samples and seawater analyzed in this study.**
(PDF)

Table S2 **Results of SIMPER analysis describing the OTUs contributing to skin-bacterial community similarity within the geographical area.**
(PDF)
