# Supplementary material for: Humpback Whale Populations Share a Core Skin Bacterial Community: Towards a Health Index for Marine Mammals?
Source: PLoS One. 2014 Mar 26;9(3):e90785. doi: 10.1371/journal.pone.0090785 (PMC3966734; doi:10.1371/journal.pone.0090785)
Supplement: Table S1 — Summary of humpback whale tissue samples and seawater analyzed in this study. (PDF) [file pone.0090785.s001.pdf]

Table S1. Summary of humpback whale tissue samples and seawater analyzed in this study.

Sample ID[1]	Sample type	Collection method	Ocean	Geographic Area	Habitat type[2]	Sex[3]	Age class[4]	Notes
CCS2009-043	Free-ranging whale	Biopsy	North Atlantic	Gulf of Maine	A	U	C	
CCS2009-059	Free-ranging whale	Biopsy	North Atlantic	Gulf of Maine	A	U	C	Calf of CCS2009-058
CCS2009-075	Free-ranging whale	Biopsy	North Atlantic	Gulf of Maine	A	U	C	Calf of CCS2009-076
CCS2009-076	Free-ranging whale	Biopsy	North Atlantic	Gulf of Maine	A	F	A	
CCS2009-084	Free-ranging whale	Biopsy	North Atlantic	Gulf of Maine	A	U	A	
CCS2009-086	Free-ranging whale	Biopsy	North Atlantic	Gulf of Maine	A	M	A	
CCS2009-090	Free-ranging whale	Biopsy	North Atlantic	Gulf of Maine	A	F	C	
CCS2009-097	Free-ranging whale	Biopsy	North Atlantic	Gulf of Maine	A	U	U	
CCS2010-96	Free-ranging whale	Biopsy	North Atlantic	Gulf of Maine	A	n/a	n/a	Microscopy only
CCS2010-97	Free-ranging whale	Biopsy	North Atlantic	Gulf of Maine	A	n/a	n/a	Microscopy only
CCS2010-98	Free-ranging whale	Biopsy	North Atlantic	Gulf of Maine	A	n/a	n/a	Microscopy only
CCS2010-99	Free-ranging whale	Biopsy	North Atlantic	Gulf of Maine	A	n/a	n/a	Microscopy only
CCS2010-100	Free-ranging whale	Biopsy	North Atlantic	Gulf of Maine	A	n/a	n/a	Microscopy only
CCS2010-101	Free-ranging whale	Biopsy	North Atlantic	Gulf of Maine	A	n/a	n/a	Microscopy only
CCS2011-001	Free-ranging whale	Biopsy	North Atlantic	Gulf of Maine	A	n/a	n/a	Microscopy only
CCS2009-044	Free-ranging whale	Sloughed	North Atlantic	Gulf of Maine	A	U	C	Same as CCS2009-043
CCS2009-057	Free-ranging whale	Sloughed	North Atlantic	Gulf of Maine	A	M	J	
CCS2009-058	Free-ranging whale	Sloughed	North Atlantic	Gulf of Maine	A	F	A	
CCS2009-062	Free-ranging whale	Sloughed	North Atlantic	Gulf of Maine	A	M	C	
CCS2009-064 (a,b)	Free-ranging whale	Sloughed	North Atlantic	Gulf of Maine	A	M	U	
CCS2009-065	Free-ranging whale	Sloughed	North Atlantic	Gulf of Maine	A	F	J	
CCS2009-066	Free-ranging whale	Sloughed	North Atlantic	Gulf of Maine	A	U	J	Calf of CCS2009-058
CCS2009-067	Free-ranging whale	Sloughed	North Atlantic	Gulf of Maine	A	U	U	
CCS2009-074	Free-ranging whale	Sloughed	North Atlantic	Gulf of Maine	A	M	A	
CCS2009-077	Free-ranging whale	Sloughed	North Atlantic	Gulf of Maine	A	F	J	
CCS2009-082	Free-ranging whale	Sloughed	North Atlantic	Gulf of Maine	A	M	J	
CCS2009-093	Free-ranging whale	Sloughed	North Atlantic	Gulf of Maine	A	F	J	
WH44	Free-ranging whale	Biopsy	North Pacific	Hawaii	B	M	A	
WH45 (a,b)	Free-ranging whale	Biopsy	North Pacific	Hawaii	B	U	A	
WH46	Free-ranging whale	Biopsy	North Pacific	Hawaii	B	U	A	
WH47	Free-ranging whale	Biopsy	North Pacific	Hawaii	B	M	A	
WH49-b	Free-ranging whale	Biopsy	North Pacific	Hawaii	B	M	A	

Table S1. Summary of humpback whale tissue samples and seawater analyzed in this study.

Sample ID[1]	Sample type	Collection method	Ocean	Geographic Area	Habitat type[2]	Sex[3]	Age class[4]	Notes
WH50	Free-ranging whale	Biopsy	North Pacific	Hawaii	B	M	A	
WH51	Free-ranging whale	Biopsy	North Pacific	Hawaii	B	M	A	
WH52	Free-ranging whale	Biopsy	North Pacific	Hawaii	B	U	C	
WH53	Free-ranging whale	Biopsy	North Pacific	Hawaii	B	F	A	
WH55	Free-ranging whale	Biopsy	North Pacific	Hawaii	B	F	A	
WH56	Free-ranging whale	Biopsy	North Pacific	Hawaii	B	M	A	
WH57	Free-ranging whale	Biopsy	North Pacific	Hawaii	B	M	A	
SEAK-1 (a,b)	Free-ranging whale	Sloughed	North Pacific	Southeast Alaska	A	U	A	
SEAK-2 (a,b)	Free-ranging whale	Sloughed	North Pacific	Southeast Alaska	A	U	A	
SEAK-3	Free-ranging whale	Sloughed	North Pacific	Southeast Alaska	A	U	A	
WH44M	Free-ranging whale	Sloughed	North Pacific	Hawaii	B	M	A	Recovered from tag
WH57BR (a,b)	Free-ranging whale	Sloughed	North Pacific	Hawaii	B	M	A	Recovered from tag, same as WH57
WH61 (a,b)	Free-ranging whale	Sloughed	North Pacific	Hawaii	B	M	A	Recovered from tag
FBNMS-09-001	Free-ranging whale	Biopsy	South Pacific	American Samoa	B	M	A	
FBNMS-09-003	Free-ranging whale	Biopsy	South Pacific	American Samoa	B	M	A	
FBNMS-09-004	Free-ranging whale	Biopsy	South Pacific	American Samoa	B	M	A	
FBNMS-09-005	Free-ranging whale	Biopsy	South Pacific	American Samoa	B	F	A	
FBNMS-09-006	Free-ranging whale	Sloughed	South Pacific	American Samoa	B	M	A	
FBNMS-09-007	Free-ranging whale	Sloughed	South Pacific	American Samoa	B	F	A	
FBNMS-09-010	Free-ranging whale	Biopsy	South Pacific	American Samoa	B	U	A	
FBNMS-09-011	Free-ranging whale	Sloughed	South Pacific	American Samoa	B	U	A	Same as FBNMS-09-010
FBNMS-09-012	Free-ranging whale	Biopsy	South Pacific	American Samoa	B	M	A	
FBNMS-09-013	Free-ranging whale	Biopsy	South Pacific	American Samoa	B	M	A	
FBNMS-09-014	Free-ranging whale	Biopsy	South Pacific	American Samoa	B	M	A	
FBNMS-09-015	Free-ranging whale	Sloughed	South Pacific	American Samoa	B	M	A	
FBNMS-09-016	Free-ranging whale	Biopsy	South Pacific	American Samoa	B	M	A	
ENT1 (a,b)	Entangled whale	Sloughed	North Pacific	Hawaii	B	M	A	Whale exhibited entanglement injuries and visual evidence of health impairment. Believed to have become entangled in Southeast Alaska at least two

Table S1. Summary of humpback whale tissue samples and seawater analyzed in this study.

Sample ID[1]	Sample type	Collection method	Ocean	Geographic Area	Habitat type[2]	Sex[3]	Age class[4]	Notes
								months earlier. Skin was recovered from gear in disentanglement.
ENT2	Entangled whale	Biopsy	North Pacific	Hawaii	B	U	A	Whale exhibited entanglement injuries and mild visual evidence of health impairment.
SxMn1023	Dead whale	Necropsy sample	North Atlantic	Gulf of Maine	A	F	J	Time since death is unknown, but assessed as "freshly dead" (Code 2) and death was thought to have been rapid (asphyxia).
IFAW10-188Mn (a,b)	Dead whale	Necropsy sample	North Atlantic	Gulf of Maine	A	M	J	Samples were collected after the carcass stranded ashore, three days after first found dead at sea. The carcass was moderately decomposed (Code 3).
Calf (a, b, c)	Dead whale	Necropsy sample	North Pacific	Hawaii	B	F	C	Died after stranding ashore alive. Samples were taken within an hour of death from below the head, dorsal portion of the head, and the peduncle area.
OWSI 2	Seawater	Grab sample	North Pacific	Hawaii	B	n/a	n/a	
OWSI 4	Seawater	Grab sample	North Pacific	Hawaii	B	n/a	n/a	
MSW 1	Seawater	Grab sample	North Pacific	Hawaii	B	n/a	n/a	
MSW 3	Seawater	Grab sample	North Pacific	Hawaii	B	n/a	n/a	

[1]a,b and c designate replicate samples
[2]A = Temperate area, feeding habitat, catabolic metabolism assumed, B=Tropical area, breeding habitat, anabolic metabolism assumed
[3]M=male, F=female, U=unknown
[4]C=dependent calf, J=independent juvenile, A = Adult, U=unknown
