# Supplementary material for: Humpback Whale Populations Share a Core Skin Bacterial Community: Towards a Health Index for Marine Mammals?
Source: PLoS One. 2014 Mar 26;9(3):e90785. doi: 10.1371/journal.pone.0090785 (PMC3966734; doi:10.1371/journal.pone.0090785)
Supplement: Table S2 — Results of SIMPER analysis describing the OTUs contributing to skin-bacterial community similarity within the geographical area. (PDF) [file pone.0090785.s002.pdf]

Table S2. Results of SIMPER analysis describing the OTUs contributing to skin-bacterial community similarity within the geographical area.

OTU	Av. Abundance	Av. Similarity	Sim/SD	Contribution to similarity (%)		Taxonomy
Gulf of Maine						
3	12.42	4.99	0.72	21.54		*Bacteroidetes, Tenacibaculum*
2	11.69	4.57	0.81	19.73		*Gammaproteobacteria, Psychrobacter*
12	3.62	1.57	0.83	6.8		*Bacteroidetes, Tenacibaculum*
10*	5.12	1.45	0.51	6.27	GOM	*Bacteroidetes, Tenacibaculum*
29	3.45	1.13	0.65	4.87		*Bacteroidetes, Tenacibaculum*
19	3.65	0.9	0.56	3.89	AK & GOM	*Gammaproteobacteria, Psychrobacter*
4	5.32	0.9	0.36	3.87		*Bacteroidetes, Flavobacteriaceae,* uncultured humpback group
28	3.07	0.89	0.48	3.84		*Gammaproteobacteria, Psychrobacter*
9	3.96	0.76	0.36	3.29		*Gammaproteobacteria, Moraxellaceae*
7	2.12	0.53	0.46	2.28		*Gammaproteobacteria, Psychrobacter*
27	1.91	0.36	0.39	1.54		*Bacteroidetes, Tenacibaculum*
5	2.85	0.35	0.55	1.5		*Gammaproteobacteria, Cardiobacteria*
11	2.19	0.35	0.33	1.49		*Gammaproteobacteria, Psychrobacter*
33	1.03	0.3	0.54	1.31	AK & GOM	*Gammaproteobacteria, Colwellia*
17	1.72	0.29	0.49	1.26	AK & GOM	*Gammaproteobacteria, Psychrobacter*
20	1.6	0.29	0.3	1.24	HI & GOM	*Alphaproteobacteria*, Roseobacter clade AS-21
51	0.89	0.28	0.6	1.21	AK & GOM	*Bacteroidetes, Tenacibaculum*
1	1.22	0.24	0.41	1.02		*Bacteroidetes, Tenacibaculum*
Alaska						
21	17.17	7.18	0.61	28.51	AK	*Gammaproteobacteria, Psychrobacter*
8	26.73	6.99	0.5	27.75	AK	*Bacteroidetes, Tenacibaculum*
33	5.81	2.57	0.8	10.2	AK & GOM	*Gammaproteobacteria, Colwellia*
3	5.11	1.61	0.53	6.4		*Bacteroidetes, Tenacibaculum*
19	7.67	1.59	0.32	6.3	AK & GOM	*Gammaproteobacteria, Psychrobacter*
28	3.96	0.87	0.34	3.47		*Gammaproteobacteria, Psychrobacter*
17	1.27	0.32	0.41	1.26	AK & GOM	*Gammaproteobacteria, Psychrobacter*
49	1.7	0.31	0.32	1.23	AK	*Gammaproteobacteria, Colwellia*
29	0.66	0.3	0.75	1.17	AK	*Bacteroidetes, Tenacibaculum*

Table S2. Results of SIMPER analysis describing the OTUs contributing to skin-bacterial community similarity within the geographical area.

OTU	Av. Abundance	Av. Similarity	Sim/SD	Contribution to similarity (%)		Taxonomy
51	1.36	0.29	0.46	1.16	AK & GOM	*Bacteroidetes, Tenacibaculum*
110	1.16	0.28	0.33	1.11	AK	*Gammaproteobacteria, Colwellia*
Hawaii						
2	14.61	4.05	0.49	23.31		*Gammaproteobacteria, Psychrobacter*
4	8.91	2.43	0.48	13.94		*Bacteroidetes, Flavobacteriaceae, uncultured humpback group*
1	12.91	1.69	0.21	9.72		*Bacteroidetes, Tenacibaculum*
7	5.03	1.03	0.33	5.91		*Gammaproteobacteria, Psychrobacter*
9	4.11	0.99	0.4	5.68		*Gammaproteobacteria, Moraxellaceae*
15	3.46	0.91	0.44	5.25	HI & Am Samoa	*Betaproteobacteria, Aquabacterium*
27	1.87	0.72	0.75	4.12		*Bacteroidetes, Tenacibaculum*
5	1.44	0.43	0.51	2.48	HI & Am Samoa	*Gammaproteobacteria, Cardiobacteria*
20	1.59	0.41	0.49	2.35	HI & GOM	*Alphaproteobacteria*, Roseobacter clade AS-21
39	1.23	0.38	0.47	2.16	HI & Am Samoa	*Gammaproteobacteria, Escherichia*
11	0.91	0.35	0.71	2		*Gammaproteobacteria, Psychrobacter*
35	1.67	0.32	0.28	1.85	HI	*Alphaproteobacteria, Novosphingobium* (aromatic hydrocarbon degrader, glycosphingolipids (sphingans) - useful for colonization of eukaryotic organisms)
12	3.38	0.31	0.45	1.77		*Bacteroidetes, Tenacibaculum*
14	4.75	0.31	0.18	1.76	HI	*Gammaproteobacteria, Psychrobacter*
American Samoa						
27	9.09	3.59	0.65	18.58		*Bacteroidetes, Tenacibaculum*
2	12.57	3.48	0.45	17.99		*Gammaproteobacteria, Psychrobacter*
3	7.68	1.6	0.38	8.25		*Bacteroidetes, Tenacibaculum*
12	7.17	1.45	0.54	7.51		*Bacteroidetes, Tenacibaculum*
11	6.24	1.13	0.54	5.82		*Gammaproteobacteria, Psychrobacter*
1	6.68	0.92	0.39	4.73		*Bacteroidetes, Tenacibaculum*

Table S2. Results of SIMPER analysis describing the OTUs contributing to skin-bacterial community similarity within the geographical area.

OTU	Av. Abundance	Av. Similarity	Sim/SD	Contribution to similarity (%)		Taxonomy
5	1.77	0.71	0.73	3.66	HI & Am Samoa	*Gammaproteobacteria, Cardiobacteria*
9	2.52	0.71	0.51	3.66		*Gammaproteobacteria, Pseudomonadales, Moraxellaceae*
7	2.73	0.7	0.4	3.6		*Gammaproteobacteria, Psychrobacter*
15	2.46	0.6	0.58	3.08	HI & Am Samoa	*Betaproteobacteria, Burkholderiales, Aquabacterium*
37	3.13	0.46	0.23	2.37	Am Samoa	*Bacteroidetes, Flavobacteriaceae*
50	1.68	0.42	0.37	2.17	Am Samoa	*Bacteroidetes, Flavobacteriaceae,* uncultured humpback group
22	3.95	0.39	0.22	2	Am Samoa	*Proteobacteria,* Incertae Sedis (humpback-specific)
41	2.66	0.36	0.22	1.84	Am Samoa	*Bacteroidetes, Flavobacteriaceae*
39	0.65	0.21	0.53	1.07	HI & Am Samoa	*Gammaproteobacteria, Escherichia-Shigella*
38	1.37	0.2	0.33	1.02	Am Samoa	*Gammaproteobacteria, Colwellia*
